# A Topical Cosmeceutical Approach to Submental Fullness in Indian Women: 12‐Week Prospective Study

**DOI:** 10.1111/jocd.70653

**Published:** 2026-01-05

**Authors:** Subashini Selavdurairaj, Savitha Murali, Sivavallinathan Arunachalam, Ragavi Balasubramanian, C. Divyalakshmi, Vanathi Thirunavukkarasu, S. Anannya, Pavithra Sukumaran

**Affiliations:** ^1^ Silphion Research Private Limited Chennai India; ^2^ Render Skin & Hair Chennai India

**Keywords:** DMAE (dimethylaminoethanol), double chin, Indian women, lower facial aging, non invasive treatment, submental fullness, topical cosmeceutical

## Abstract

**Background:**

Submental fullness (“double chin”) is a common aesthetic concern among Indian women. No topical cosmeceutical has been systematically evaluated for this indication.

**Objective:**

To assess the efficacy of a multi‐active topical serum containing DMAE and retinol in reducing submental fullness in Indian women.

**Methods:**

This 12‐week, single‐center, open‐label study enrolled 16 women (ages 18–60) with mild‐to‐moderate submental fullness; 13 completed the trial. Subjects applied the serum nightly to the submental area. Standardized photographs were obtained at baseline and week 12 and were independently graded by two dermatologists using the validated Clinician‐Reported Submental Fat Rating Scale. Any discordance was evaluated and resolved by a third, umpire dermatologist. Body weight was monitored throughout.

**Results:**

All 13 completers showed visible improvement in submental contour. Dermatologist grading showed statistically significant improvements across all three views (*p* < 0.05), with the maximum change noted in the left and front profiles (*r* = 0.88 and 0.83, respectively) compared to the right profile (*r* = 0.61). Improvements were independent of body weight changes. No adverse events occurred.

**Conclusion:**

This preliminary study demonstrates that a DMAE and retinol based topical serum can improve submental fullness in Indian women over 12 weeks, offering a noninvasive alternative to procedural interventions. Larger randomized controlled trials with objective volumetric endpoints are needed for validation.

## 1 Introduction

Submental fullness, or “double chin,” is a well‐recognized aesthetic concern with documented negative effects on quality of life, particularly in women [1]. This issue holds special relevance for Indian women, who often exhibit a hypertrophic aging pattern marked by central facial volume accumulation and early submental convexity, in contrast to Caucasian cohorts who more commonly demonstrate deflationary changes with sagging as a secondary component [2].

Evidence from Asian populations reveals that skin elasticity decline begins as early as age 25, with horizontal neck folds typically appearing between ages 26–30 [3, 4]. This early onset of aging changes predisposes Indian women to earlier and more visible submental fullness, amplifying dissatisfaction with facial appearance and highlighting the critical need for accessible preventive interventions.

The pathophysiology of lower facial aging is multifactorial, involving both intrinsic factors (chronological and genetic) and extrinsic factors including sun exposure, air pollution, smoking, diet, and repetitive mechanical stress. Modern lifestyle factors have amplified these concerns—“tech neck,” characterized by repeated flexing and chin lowering during prolonged device use, accelerates fold formation and skin laxity in younger populations. The visible manifestations include loss of elasticity with descent of jowl fat, laxity of neck skin and underlying structures, perioral retrusion, chin remodeling, and progressive jawline blunting. Collectively, these changes erode the sharp mandibular contour and cervicomental angle that define a youthful profile [4]. At the tissue level, degradation of the dermal extracellular matrix (ECM), specifically collagen and elastin breakdown, combined with gravitational effects on superficial fat compartments, drives the characteristic loss of definition seen in lower facial aging [5].

Current treatment options include surgical interventions and device‐based modalities such as radiofrequency, focused ultrasound, cryolipolysis, deoxycholic acid injections, botulinum toxin, and light based devices. However, these approaches require clinical visits, carry procedural risks and downtime, involve significant cost, and often necessitate combination protocols for optimal results. Meanwhile, the rise of video conferencing and social media, the so‐called “Zoom Effect” [6] has intensified awareness of the lower face and neck regions, particularly among younger populations seeking accessible, non‐invasive solutions. Despite these insights into lower facial aging, no topical cosmeceutical has been systematically evaluated for addressing submental fullness in Indian women. We present a novel non‐invasive cosmeceutical approach to this aesthetic concern.

## 2 Methods

### 2.1 Study Design

The trial was conducted at a single center using an open‐label design over 12 weeks to evaluate a multi‐active topical serum for mild to moderate submental fullness in Indian women. Ethics committee approval was obtained for the protocol, and all participants provided written informed consent before enrollment.

### 2.2 Study Population

Sixteen healthy Indian women aged 18–60 years were enrolled in the study. Eligible participants were required to have clinician‐graded mild to moderate submental fullness and stable body weight for a minimum of 3 months prior to enrollment. Participants agreed to use only the study serum on the submental area and refrain from applying other firming or contouring products throughout the study duration.

Exclusion criteria included pregnancy or lactation, active dermatologic conditions in the treatment area, and any known hypersensitivity to formulation ingredients. Subjects who had undergone minimally invasive cosmetic treatments such as botulinum toxin, dermal fillers, or energy‐based devices in the lower face or neck region within 6 months were also excluded.

### 2.3 Study Protocol

#### 2.3.1 Intervention

The intervention consisted of a novel cosmeceutical serum (Sculpt Serum, CHOSEN), containing dimethylaminoethanol (DMAE), retinol, methylsulfonylmethane (MSM), and beet extract as primary active ingredients. The formulation was designed to target dermal ECM integrity and skin mechanical properties through complementary mechanisms of action.

#### 2.3.2 Application Protocol

Participants applied the serum at bedtime, to the submental region and jawline following a standardized application method. Prior to application, the treatment area was cleansed. Subjects were instructed to avoid massage techniques, aesthetic devices, or additional firming or contouring products in the treatment area throughout the 12‐week study duration. Regular photoprotective measures were maintained as part of participants' standard skincare routines.

### 2.4 Assessment Methods

Outcomes were assessed at baseline and Week 12. Standardized digital photography was performed at each visit under fixed lighting conditions and camera settings, capturing frontal, right and left profiles. The outcome was independently graded by two dermatologists using the validated Clinician Reported Submental Fat Rating Scale (0 = none; 1 = mild; 2 = moderate; 3 = moderately severe; 4 = severe) [7]. Any discordance was evaluated and resolved by a third, umpire dermatologist. Body weight was measured at each assessment visit. Tolerability was evaluated through clinician assessment and participant diaries documenting any adverse events or product‐related concerns.

### 2.5 Data Management and Statistical Analysis

Study data were securely coded, digitally entered, and stored confidentially in accordance with institutional protocols. For comparisons between baseline and post‐intervention values, the Wilcoxon signed‐rank test was used. Repeated measures analysis of covariance (ANCOVA) was applied to assess the effect of the intervention independent of any changes in body weight. Effect sizes were calculated using r values. Analyses were conducted using IBM SPSS Statistics version 29.0.2.0, with statistical significance set at *p* < 0.05.

## 3 Results

Of the 16 women enrolled, 13 completed the 12‐week study; three participants were unable to attend the final assessment visit. No adverse events were reported throughout the study period.

**FIGURE 1:**
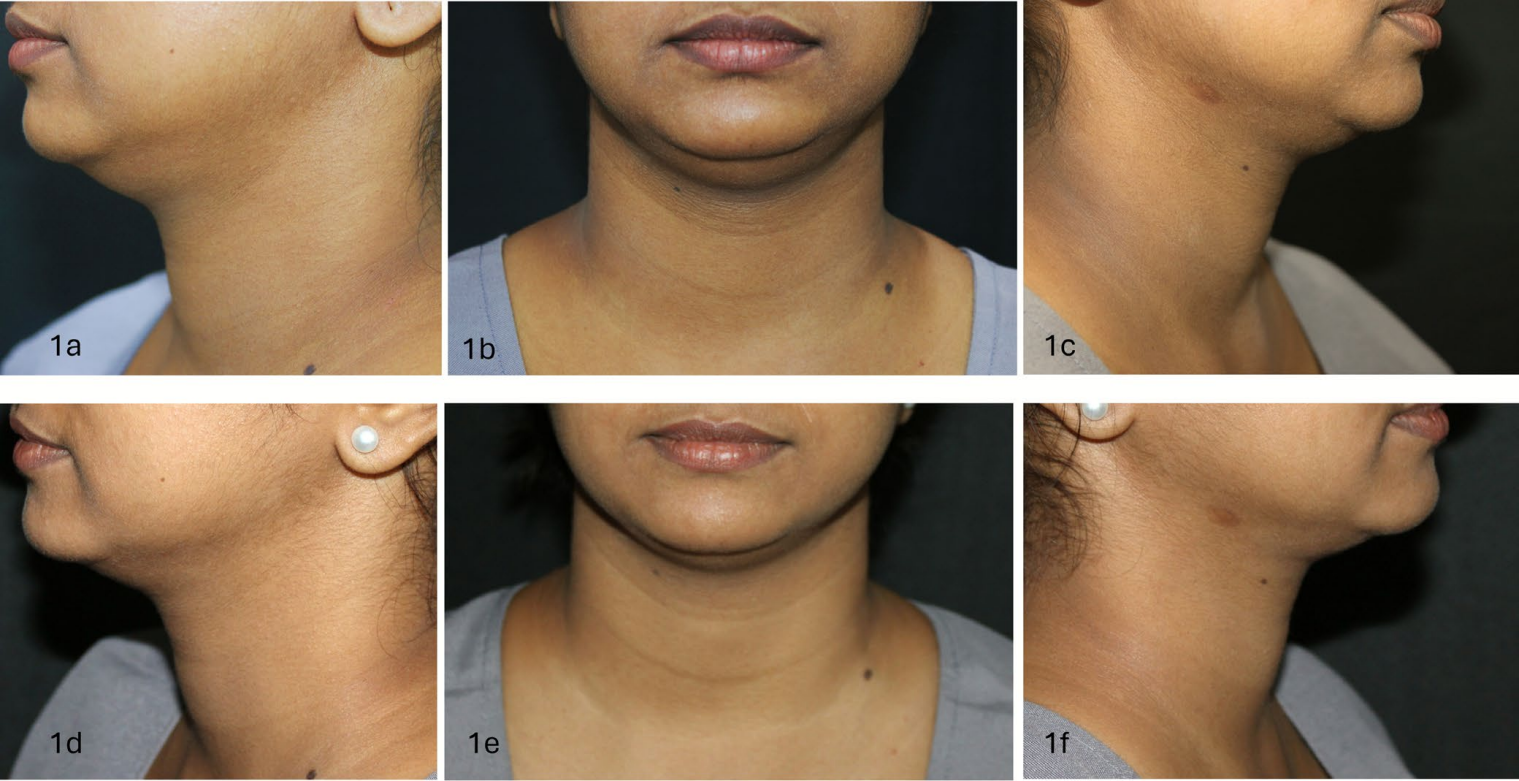
Standardized photographs of front, left and right face views at baseline and at end of 12 weeks. (a) Left face view at baseline of patient 1. (b) Front face view at baseline of patient 1. (c) Right face view at baseline of patient 1. (d) Left face view at the end of 12 weeks of patient 1. (e) Front face view at the end of 12 weeks of patient 1. (f) Right face view at the end of 12 weeks of patient 1.

**FIGURE 2:**
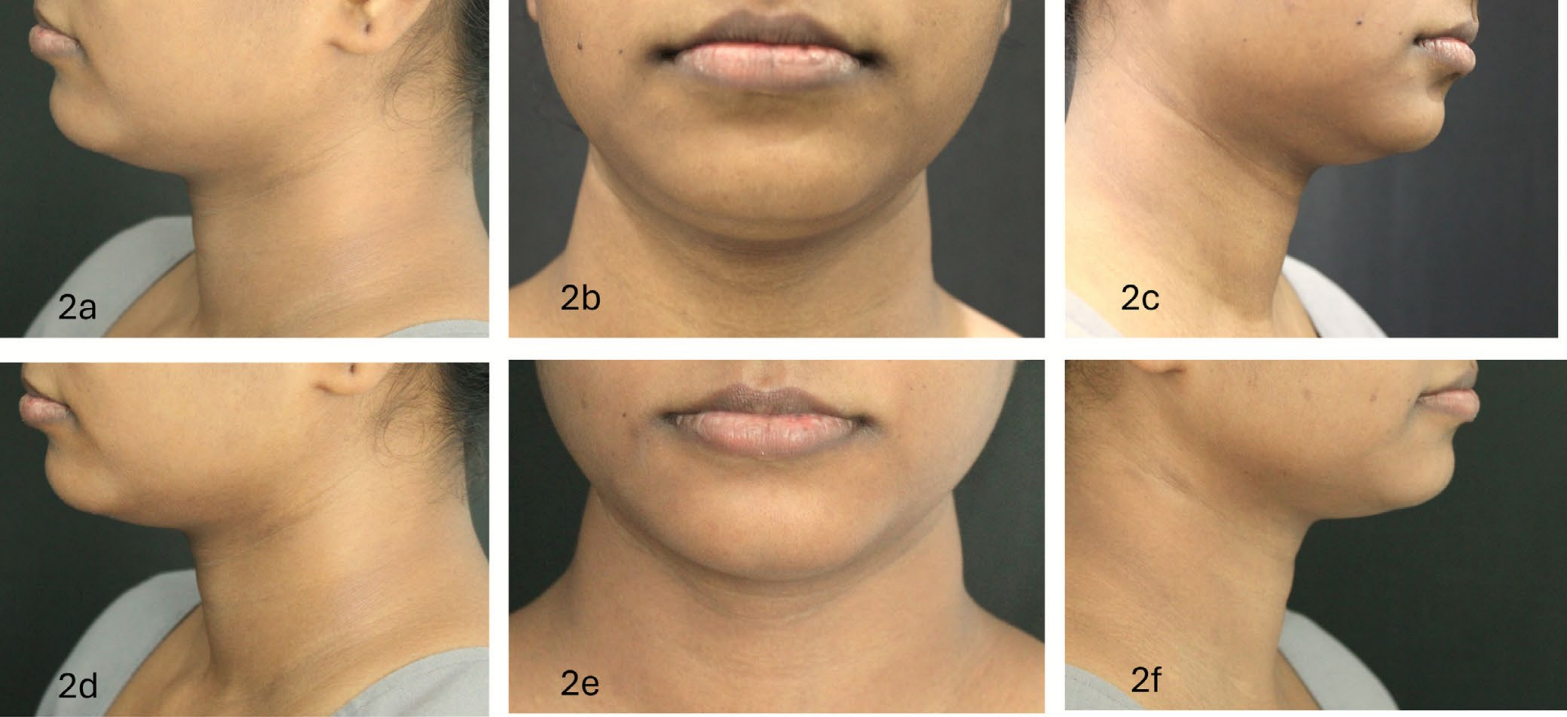
Standardized photographs of front, left, and right face views at baseline and at end of 12 weeks—PATIENT 2. (a) Left face view at baseline of patient 2. (b) Front face view at baseline of patient 2. (c) Right face view at baseline of patient 2. (d) Left face view at the end of 12 weeks of patient 2. (e) Front face view at the end of 12 weeks of patient 2. (f) Right face view at the end of 12 weeks of patient 2.

All 13 completers demonstrated visible improvement in submental contour, with 69% showing an improvement in at least two views (Figures 1 and 2). Dermatologist grading showed statistically significant improvements across all three views (*p* < 0.05), with the maximum change noted in the left and front profiles (*r* = 0.88 and 0.83, respectively) compared to the right profile (*r* = 0.61). ANCOVA confirmed that observed improvements were independent of body weight changes, with no significant effect of weight variation on visual scores across all views (*p* > 0.05 for all comparisons).

All participants tolerated the serum, without any dryness, redness, or flaking. No other adverse events were reported.

## 4 Discussion

Submental fullness presents a significant aesthetic concern that has historically required invasive surgical interventions or injectable treatments with associated risks and downtime. This 12‐week study demonstrates that a DMAE‐based topical formulation produces measurable improvements in submental fullness in Indian women, offering a non‐invasive alternative.

The formulation addresses multiple aging pathways. DMAE increases dermal thickness and enhances collagen fiber organization [8, 9, 10], while retinol drives epidermal renewal and collagen synthesis [11]. MSM [12] and beet extract provide photoprotective antioxidant activity and hydration. Consistent with this multi target approach, all 13 study completers showed visible improvement, with statistically significant reductions across all photographic views (*p* < 0.05).

Majority of the women noted a significant and visible change in at least two views, as demonstrated by the large effect sizes. Improvements were independent of weight changes, confirming effects on submental tissue structure rather than systemic fat loss.

The results are particularly relevant for Indian women, who exhibit hypertrophic aging with early submental convexity. Evidence shows skin elasticity decline begins at age 25 in Asian populations, with neck folds appearing by ages 26–30 [3, 4]. Our cohort (ages 18–60) demonstrates the dual utility of topical intervention, preventive for younger patients and corrective for established concerns.

Compared to injectable treatments for submental fat such as deoxycholic acid, which are associated with swelling, bruising, discomfort, and procedural risks including nerve damage [13], the topical approach provides an accessible alternative with visible improvement on the Clinician‐Reported Submental Fat Rating Scale as assessed by blinded dermatologists, with no adverse events. Additionally, Indian aesthetic preferences favor non invasive treatments more than Western markets where injectables dominate, making topical solutions culturally attractive [14]. The rise of “tech neck” and video conferencing has further amplified demand for accessible lower facial treatments [4].

Study limitations include small sample size (*n* = 13), open‐label design, absence of controls, and reliance on photographic rather than volumetric assessment. The 12‐week duration may not capture full long‐term effects. Future studies should incorporate 3D imaging and elastometry, particularly since DMAE requires dermal penetration for collagen‐enhancing effects. Age‐stratified analysis to identify optimal candidates and larger randomized controlled trials are warranted.

## 5 Conclusion

This study provides preliminary evidence that this DMAE‐based topical serum visibly improves submental contour through documented effects on dermal structure and collagen organization. For Indian women experiencing early hypertrophic aging, this accessible, non‐invasive approach addresses both prevention and correction without the risks of procedural interventions. The formulation represents a practical option aligned with contemporary aesthetic needs and cultural treatment preferences.

## Author Contributions

All the authors have contributions in this study.

## Ethics Statement

The authors have nothing to report.

## Consent

Obtained from all the volunteers.

## Conflicts of Interest

The authors declare no conflicts of interest.

## Data Availability

The data that support the findings of this study are available from the corresponding author upon reasonable request.
